# Immediate Surgery Might Be a Better Option for Subcapsular Thyroid Microcarcinomas

**DOI:** 10.1155/2019/3619864

**Published:** 2019-04-03

**Authors:** Jun Jiang, Hui Lu

**Affiliations:** Department of General Surgery, the First Affiliated Hospital of Nanjing Medical University, No. 300 Guangzhou Road, Nanjing 210029, China

## Abstract

For high-risk papillary thyroid microcarcinomas (PTMCs), immediate surgery is recommended. This study aimed to evaluate the location of PTMCs in the thyroid lobe and determine whether location is associated with an aggressive biology and the necessity of immediate surgery. This retrospective study included 288 patients who underwent initial surgery for PTMC. Clinical data were extracted. Subcapsular thyroid microcarcinomas (STMs) and nonsubcapsular thyroid microcarcinomas (NSTMs), distinguished by ultrasound, were compared in terms of tumour size, extrathyroidal extension, cervical lymph node metastasis, and multifocality. The preoperative ultrasound features predictive of recurrent laryngeal nerve (RLN) involvement were assessed. There were no statistical differences in tumour size (*P* = 0.985), multifocality (*P* = 0.866), lymph node metastases to the central compartment (*P* = 0.154), or lateral lymph node metastases (*P* = 0.929) between STM and NSTM groups. Macroscopic extrathyroidal extension was exclusively found in the STM group. For assessing RLN involvement, the sensitivity, specificity, and positive predictive value of the presence of an abnormal thyroid capsule margin between the STM and the presumed RLN course, assessed on preoperative ultrasonography images, were 100%, 43.3%, and 43.3%, respectively. Immediate surgery appears to be a better option than conservative treatment for these high-risk STM patients.

## 1. Introduction

Papillary thyroid carcinoma (PTC) is the most common well-differentiated thyroid carcinoma. The majority of newly diagnosed PTCs are papillary thyroid microcarcinomas (PTMCs), defined as PTC ≤ 1 cm in maximal diameter [1, 2], primarily due to the routine use of high-resolution ultrasonography in regular health examinations [3]. Many researchers have recommended conservative management for low-risk PTMCs due to their slow growth [4]. It is worth noting that the definition of PTMC is PTC with a maximal diameter of ≤1 cm, regardless of its biological behaviour. PTMCs with cervical lymph node metastasis (LNM) and locoregional invasiveness or recurrence are frequently detected [5]. Less frequently, PTMCs with distant metastases are reported [6]. It is reasonable to identify and treat high-risk PTMCs with aggressive features. Nevertheless, PTMCs located close to the capsular area, identified by radiological investigations, should also be considered serious. Subcapsular thyroid microcarcinomas (STMs) can easily progress to extrathyroidal extension (ETE), possibly invading important surrounding tissues such as the trachea, recurrent laryngeal nerve (RLN), or carotid artery; however, they are not necessarily associated with biological aggressiveness. For these STMs, individualised treatment strategies including active surveillance, lobectomy, or total thyroidectomy with or without cervical lymph node dissection should be carefully considered [2, 7].

The purpose of this study was to investigate the clinical and ultrasonography characteristics of STMs and to determine whether they are associated with aggressive behaviour.

## 2. Materials and Methods

### 2.1. Patient Selection

Patients with PTMC who underwent initial thyroid surgery between June 2017 and June 2018 were included in this retrospective, single-centre study. All these patients were diagnosed as PTMC on ultrasonography-guided fine needle aspiration biopsy (FNAB).

PTMC patients without clinical lymph node or distant metastasis, who were managed with observation, were excluded. Patients with PTMC of the thyroid isthmus or incidentally detected PTMC during surgery for benign thyroid disease were also excluded.

### 2.2. Clinical Data

The medical records of all patients with pathologically confirmed PTMC were reviewed. Individualised surgery strategies were based on preoperative sonographic evaluation, intraoperative inspection, and frozen section. All patients underwent routine prophylactic central neck dissection. Therapeutic lateral cervical neck dissection was indicated in patients with clinically apparent lateral cervical LNM. Preoperative laryngoscopy was routinely performed in all patients. Postoperative laryngoscopy was performed in symptomatic patients with voice change on postoperative day 1.

### 2.3. Preoperative Imaging

**Figure 1:**
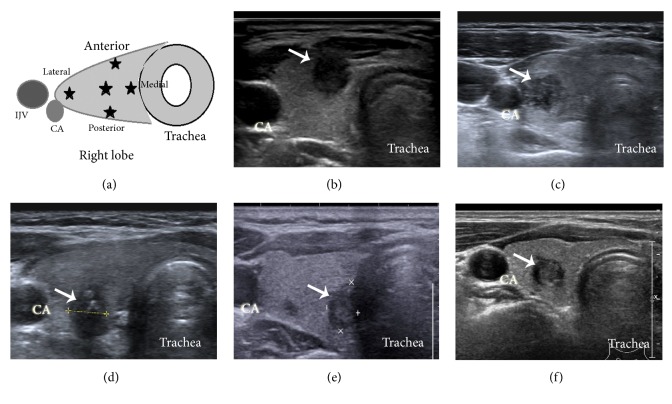
(a) Schematic of different locations (asterisks) of thyroid microcarcinomas in the thyroid lobe. Subcapsular thyroid microcarcinomas (arrow) can be located (b) anterior to the strap muscles, (c) lateral to the carotid artery, and (d) posterior or (e) medial to the trachea in the lobe. Nonsubcapsular thyroid microcarcinomas are located in the middle of the lobe at a certain distance from the thyroid capsule. CA, carotid artery; IJV, internal jugular vein.

Representative transverse and longitudinal ultrasonography images in the database were reviewed by an expert ultrasonography radiologist and a surgeon. STM was defined as a nodule bordering or already penetrating the thyroid capsule, or even with obvious ETE. If it did not meet these criteria, intrathyroidal microcarcinoma was defined as a nonsubcapsular thyroid microcarcinoma (NSTM) (Figure 1). Some researchers have previously noted that PTC along the RLN course may be more likely to invade the RLN. Whether STM invades the RLN is judged according to the presence of a normal margin of the thyroid capsule between the STM and the presumed location of the RLN on ultrasound images [8]. The possibility of local invasion by STM was also investigated in this study.

### 2.4. Statistical Analyses

Comparisons between two groups were made using Wilcoxon signed-rank test, chi-square test, or Fisher's exact test. The sensitivity, specificity, positive predictive value, and negative predictive value were evaluated for the predictive value of sonographic ETE features in RLN invasion. Any* P*-value ≤ 0.05 was considered to indicate a statistically significant difference.

## 3. Results

### 3.1. Baseline Characteristics of the Study Population

In the current study, initial surgery was performed on a total of 288 patients with PTMC, among which 93 patients had STMs and 195 had NSTMs. The clinical characteristics of STMs and NSTMs are presented in Table 1. STMs accounted for 93 (32.3%) of 288 PTMCs. Among these STMs, 39 were located close to the anterolateral surface (Figures 1(b) and 1(c)) of the thyroid lobe (22 in the right lobe and 17 in the left lobe), 43 were located close to the dorsal surface (Figure 1(d)) of the thyroid lobe (20 in the right lobe and 23 in the left lobe), and 11 were located close to the medial surface (Figure 1(e)) of the thyroid lobe (six in the right lobe and five in the left lobe). In both groups, the majority of PTMC patients were relatively young (under 50 years old) women. There were no cases of distant metastases in these included patients. The median tumour size revealed by histological analysis was 0.7 cm (interquartile range 0.5–0.8) in the STM group and 0.7 cm (interquartile range 0.6–0.8) in the NSTM group. There was no statistical difference in tumour size between the two groups (Wilcoxon signed-rank test,* P* = 0.985).

### 3.2. Association between Subcapsular Location and Extrathyroidal Extension

It is still unknown whether STMs are associated with aggressive biological behaviour, indicated by characteristics including ETE and cervical LNM [5]. In this study, STMs showed a significantly higher proportion of microscopic ETE (67/93, 72%) than NSTMs (0/195, 0%; Figure 1(f)). Of these STMs with microscopic ETE, 35 lesions were located in the right thyroid lobe, among which 14, 17, and 4 were located dorsally, anterolaterally, and medially to the thyroid capsule, respectively. There were 32 microscopic ETE lesions located in the left thyroid lobe, among which 16, 12, and 4 were located dorsally, anterolaterally, and medially to the thyroid capsule, respectively.

**Figure 2:**
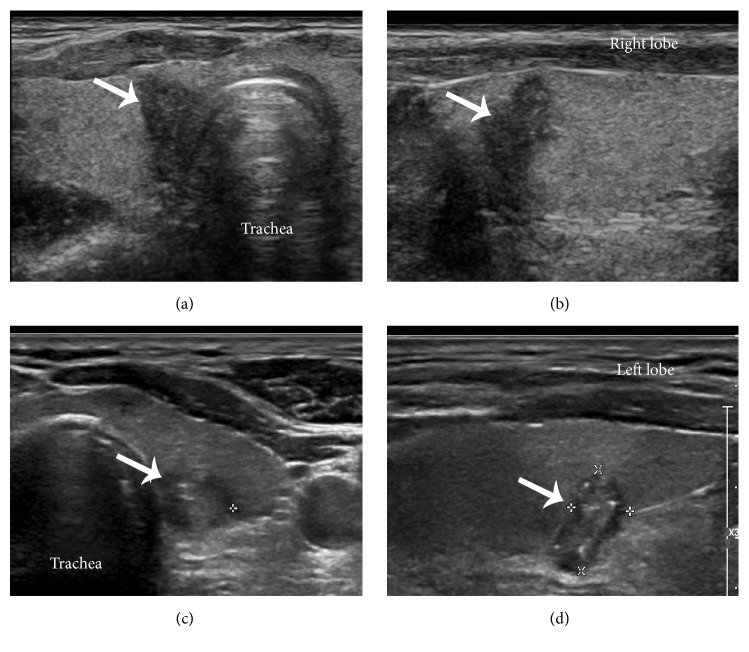
(a) Transverse and (b) longitudinal images showing a thyroid microcarcinoma (arrow) located medial to the trachea and (c) transverse and (d) longitudinal images showing a thyroid microcarcinoma located posterior to the dorsal thyroid capsule.

Gross ETE (45.2%) in STMs was confirmed by intraoperative inspection (Fisher's exact test,* P* < 0.001). The sternothyroid muscle was invaded in 22 STMs (12 in the right lobe and 10 in the left lobe), and the trachea was invaded in six STMs (three in each lobe). The RLN was involved in 15 patients (six in the right lobe and nine in the left lobe), in which preoperative laryngoscopy found no vocal cord paralysis. In the right lobe, one STM measuring 5 mm in diameter located medial to the surface of the thyroid lobe simultaneously invaded the trachea and RLN near the Berry's ligament (Figure 1(e)). One STM measuring 6 mm in diameter located medial to the trachea invaded the entry point of the RLN to the larynx without invading the trachea (Figures 2(a) and 2(b)).

### 3.3. Predictive Factors for Extrathyroidal Extension to the Recurrent Laryngeal Nerve

In the 43 STMs located close to the dorsal surface of the thyroid lobe, histological analysis identified 30 microscopic ETE lesions which lacked the normal thyroid capsule margin between the STM and the presumed RLN course on ultrasonography images (Figures 2(c) and 2(d)). Thirteen out of these 30 STMs with microscopic ETE showed gross invasion of the RLN (five in the right lobe and eight in the left lobe). These 13 STMs with RLN involvement presented a significantly larger tumour diameter (0.8 ± 0.2 cm) than the remaining 17 STMs without RLN invasion (0.5 ± 0.2 cm; Wilcoxon signed-rank test,* P* = 0.0003). Regarding ultrasonography features, the sensitivity, specificity, positive predictive value, and negative predictive value of an abnormal margin for RLN involvement were 100%, 43.3%, 43.3%, and 100%, respectively.

In most cases involving the RLN, the RLN could be easily isolated from the thyroid capsule as the tumour foci were merely in close contact with the RLN, with minimal invasion. Some STMs without dense adhesion with the RLN could be isolated by sharp dissection using mosquito forceps or scissors in order to leave as little remaining tumour as possible. No cases required partial dissection of the RLN. However, 3 out of 13 patients developed transient unilateral vocal cord paralysis, which recovered in 2–4 months.

### 3.4. Association between Subcapsular Location and Aggressive Biological Behaviour

Routine central compartment lymph node clearance in all included patients revealed no significant differences in the incidence of LNM between the STM group (37/93) and the NSTM group (61/195; chi-square test,* P* = 0.154). With respect to the incidence of lateral cervical LNM, there were also no significant differences between the STM group (5/93) and the NSTM group (10/195; chi-square test,* P* = 0.929; Table 1). In the NSTM group, a case of lymph node skip metastasis was detected.

Tumour multifocality might suggest a high-grade malignancy, which is associated with a higher risk of persistent or recurrent disease [9, 10]. In the included patients, there were no significant differences in multifocality between the STM (16/93) and NSTM (32/195) groups (chi-square test,* P* = 0.866). Twelve (12.9%) of the 93 patients in the STM group and 36 (18.5%) of the 195 patients in the NSTM group had bilateral disease, and there were also no significant differences in the bilateral distribution of tumour foci between the two groups (chi-square test,* P* = 0.237).

Some reports argue that PTC patients with coexistent Hashimoto's thyroiditis (HT) might have less aggressive disease and better clinical outcomes [11]. Thus, the proportion of patients with coexisting HT may influence the comparison of the biological behaviour between the two groups in this study. Here, we found no significant differences in the proportion of patients with coexisting HT between the STM (24/93, 25.8%) and NSTM (59/195, 30.3%) groups (chi-square test,* P* = 0.436).

## 4. Discussion

In the current study, our data indicate that STMs have a unique tendency to penetrate the thyroid capsule and invade the surrounding tissue. However, the most plausible explanation for this phenomenon is that these STMs are peripherally located, as it did not seem to be associated with an aggressive biology.

PTMCs exhibit a broad range of biological behaviours, ranging from passive to very aggressive [12, 13]. Accordingly, individualised strategies should be used to treat PTMC [2]. Nonoperative active surveillance of PTMCS is now an accepted alternative to immediate surgery [2, 14, 15]. However, Chinese patients' willingness plays an important role in treatment decision-making; thus FNAB and subsequent surgery were performed at the patient's request in many PTMC patients for fear of the potential aggressiveness of PTMC in China regardless of recommendations from some guidelines or expert consensus [15–18]. There is also no denying that very limited information is available on preoperative precise prediction of biological behaviour even with the aid of FNA-based cytological and molecular detection [3, 19–22]. Controversy still continues regarding the best treatment for PTMC patients among thyroid surgeons in China [17]. Our data showed that there were no significant differences in tumour size, multifocality, or the incidence of LNM (lateral and central lymph node metastases) between STMs and NSTMs. We included PTMC patients with LNM in order to explore whether STMs or NSTMs had a more aggressive biological behaviour based on their tendency for metastasis to central and lateral cervical lymph nodes. This result may, to some extent, indicate that there were no differences in biological behaviour between the two groups. However, significant differences in microscopic and macroscopic ETE were present between the two groups. When in contact with the thyroid capsule, STMs can easily develop into microscopic ETE tumours, not only because they have a natural regional advantage, but also because there are no reliable anatomical criteria for a pathologist to define minimal ETE due to the absence of a well-defined thyroid capsule. Previous reports indicate that microscopic ETE is not a high risk factor and is not correlated with the rates of disease-specific survival and recurrence-free survival in patients with small PTC [23, 24]. In the 8th edition of the tumour-node-metastasis classification, the microscopic ETE was no longer considered as criterion for the definition of T3 disease, while gross extrathyroidal extension as an unfavourable prognostic factor was underlined [25]. Gross ETE which develops from microscopic ETE can result in local invasion to important organs or tissues such as the trachea, RLN, oesophagus, or even the carotid artery, which further complicates surgery and worsens the prognosis of thyroid cancer [26]. Thus, individualised treatment plans should be determined based not only on the biological characteristics of PTMC, but also on its location in the thyroid lobe.

Immediate surgery is not necessary for PTMCs less than 7 mm in diameter, defined as low risk for trachea or RLN invasion in a study by Ito et al. [8]. Consistent with their results, in which the size of the PTMC was found to play an important role in RLN involvement, our data showed that posterior STMs invading the RLN were larger than those that did not invade the RLN. Different from the report by Ito et al., in the current study RLN was found to be involved in 34.9% of 43 STMs located posterior to the thyroid dorsal capsule, some of which were less than 7 mm in diameter. Fortunately, our data showed that the “invaded” RLN could be easily isolated from PTMCs by sharp dissection. In our experience, the precise identification and exposure of the RLN during surgery is useful to differentiate tumour invasion from close contact or compression, thus avoiding an arbitrary decision to sacrifice the RLN in patients with preoperative normal vocal function. Furthermore, consistent with previous reports [27, 28], a little remnant tumour tissue along the course of the preserved RLN in patients with preoperative normal vocal cord function did not increase local recurrence or mortality with the aid of postoperative radioiodine treatment based on our experience.

In addition, our data indicate that medial STMs located close to the Berry's ligament were at higher risk for RLN and trachea involvement. In the case of STMs located near the Berry's ligament or the entry point of the RLN into the larynx, even tumours with a 5 mm diameter can invade the RLN and trachea. It is difficult to remove tumour completely without damage to the RLN due to limited space for dissection. Thyroid surgeons should pay adequate attention to these PTMCs locating in this special site during operation, regardless of tumour size. It should be noted that the tumour diameters recorded in this study were obtained from pathological examination, which are generally smaller than those obtained by ultrasound measurement. The left RLN seems to be more vulnerable than the contralateral side. This can be partly explained by anatomy, as the left RLN is in a more typical medial tracheoesophageal groove position compared to the right RLN [29]. Thus, STMs, especially relatively larger ones, are not suitable for conservative treatment. In comparison of previous reports [12, 14, 30], our study further elucidated what kind of PTMCs were not suitable for active surveillance.

In our study, whether STMs involved the RLN was judged by distortion or a long interface between the STM and the thyroid capsule on the ultrasonography images [2]. Here, the sensitivity and specificity values for predicting RLN involvement based on the presence of an abnormal margin of the thyroid capsule on the sonography image were 100% and 43.3%, respectively. However, this result should be interpreted with caution, as the ultrasonography images assessed in this retrospective study were not dynamic, serially recorded, and user-dependent. This represents one of the limitations of this study. Real-time ultrasound, providing complete information, would be more reliable to confirm the presence of an abnormal margin. Moreover, variations in the course of RLN and the point or plane of STMs penetration should also be considered when evaluating the possibility of RLN involvement using the index of an “abnormal rim” by the previous report. Therefore, it is still difficult to judge RLN invasion solely by the presence of an “abnormal rim.” Another limitation of the current study is that we did not explore the specific histological subtypes of PTMC (e.g., tall cell, insular, columnar cell, and diffuse sclerosing carcinomas), which may behave more aggressively [31], between STM and NSTM groups, because these uncommon pathologic subtypes were not routinely analysed and reported in our pathology department.

## 5. Conclusions

In summary, STMs may not be associated with aggressive biological characteristics, but instead with a high risk of ETE to important adjacent structures such as the RLN, trachea, and strap muscles. Given the good prognosis of PTMC, lowering the morbidity and improving the patient's quality of life are necessary considerations of thyroid surgeons. High-risk STM patients are not candidates for conservative treatment, and active surgery appears to be a safer and better option.

## Figures and Tables

**Table 1:** Characteristics of the study subjects.

	*STM* (N = 93)	*NSTM* (N = 195)	*P value*
	Number	Number	
Age (year)	43 (35 – 42)	41 (33 – 49)	0.267 ^a^
Male	23 (24.7%)	45 (23.1%)	0.757 ^b^
Size (cm)	0.7 (0.5 - 0.8)	0.7 (0.6 - 0.8)	0.985 ^a^
Microsciopic-ETE	67 (72.0%)	0	≤ 0.001 ^c^
Macroscopic-ETE	42 (45.2%)	0	≤ 0.001 ^c^
Sternothyroid muscle	22	0	≤ 0.001 ^c^
Trachea	6	0	0.001 ^c^
RLN	15	0	≤ 0.001 ^c^
Lymph node metastasis			
N1a	37 (39.8%)	61 (31.3%)	0.154 ^b^
N1b	5 (5.4%)	10 (5.1%)	0.929 ^b^
Tumor multifocality	16 (17.2%)	32 (16.4%)	0.866 ^b^
Bilateral lesions	12 (12.9%)	36 (18.5%)	0.237 ^b^
Hashimoto's thyroiditis	24 (25.8%)	59 (30.3%)	0.436 ^b^

STM, subcapsular thyroid microcarcinoma; NSTM, nonsubcapsular thyroid microcarcinoma; ETE, extrathyroidal extension; RLN, recurrent laryngeal nerve; N1a, metastases to central cervical lymph nodes; N1b, metastases to lateral neck lymph nodes.

Continuous data are presented as the median with interquartile ranges.

^a^ Determined using the Wilcoxon signed-rank test.

^b^ Determined using the Chi-square test.

^c^ Determined using the Fisher's exact test.

## Data Availability

Data supporting this research article are available upon request.
